# Myotonic Dystrophies: State of the Art of New Therapeutic Developments for the CNS

**DOI:** 10.3389/fncel.2017.00101

**Published:** 2017-04-20

**Authors:** Genevieve Gourdon, Giovanni Meola

**Affiliations:** ^1^Institut National de la Santé et de la Recherche Médicale UMR1163Paris, France; ^2^Laboratory CTGDM, Institut Imagine, Université Paris Descartes—Sorbonne Paris CitéParis, France; ^3^Department of Biomedical Sciences for Health, Policlinico San Donato (IRCCS), University of MilanMilan, Italy

**Keywords:** myotonic dystrophy, trinucleotide repeat diseases, DM CNS symptoms, therapeutic strategies, animal models

## Abstract

Myotonic dystrophies are multisystemic diseases characterized not only by muscle and heart dysfunction but also by CNS alteration. They are now recognized as brain diseases affecting newborns and children for myotonic dystrophy type 1 and adults for both myotonic dystrophy type 1 and type 2. In the past two decades, much progress has been made in understanding the mechanisms underlying the DM symptoms allowing development of new molecular therapeutic tools with the ultimate aim of curing the disease. This review describes the state of the art for the characterization of CNS related symptoms, the development of molecular strategies to target the CNS as well as the available tools for screening and testing new possible treatments.

## General features and mechanisms

Myotonic dystrophies (DM1 and DM2) are autosomal dominant multisystemic diseases (Harper, 2001). Both are associated not only with myotonia and progressive muscle weakness, but also with broad clinical features including heart conductions defects and central nervous system (CNS) alterations. DM type 1 and type 2 are highly variable in term of age at onset, severity of the symptoms, clinical pictures, both in and between the two types. Anticipation, with aggravation of the disease severity and earlier age at onset through successive generations, is particularly evident in DM1 that can affect adults and children at birth (congenital DM1, CDM) or during childhood. Identification in 1992 of the mutation underlying DM1 in the *DMPK* gene on chromosome 19 revealed, at least in part, clues to understand this particular mode of transmission of the disease. The very highly unstable CTG repeat expansion involved in DM1 usually increases from one generation to the next and is more or less associated with the disease severity (Harper, 2001). Patients with DM1 can be divided into five main categories, each presenting specific clinical features and management problems: congenital, childhood-onset, juvenile, adult-onset, and late-onset/asymptomatic. The five clinical forms distinguish from each other by the prevalence of the main symptoms as their apparition profiles. This new classification appears to be useful in the Context of emerging therapeutic approaches and in harmonization of international myotonic dystrophy network (IDMC—International Myotonic Dystrophy Consortium; De Antonio et al., 2016). Table 1 summarizes these subtypes:

**Table 1:** **Summary of DM1 main clinical phenotypes and CNS related symptoms**.

**Phenotypes**	**Age of onset**	Clinical findings^*^	CNS symptoms^*^	**CTG length**
Congenital	Birth	Infantile hypotonia Respiratory failure Feeding difficulties Cardiorespiratory complications	Learning disability Reduced IQ values Speech and language delay	>1,000
Childhood onset	1–10 years	Facial weakness Myotonia Heart Conduction defects	Reduced IQ values Attention deficit Deficit in visuo-spatial/constructive skills Autism spectrum disorder Communication problems Social anxiety Fatigue	50–1000
Juvenile	10–20 years	Classical motor and heart symptoms can be absent and can appear later on	Visuo-spatial deficit Executive dysfunction Learning difficulty Social and Mating problems	50–1000
Adult onset	20–40 years	Weakness Myotonia Cataracts Conduction defects Insulin resistance Respiratory failure	Lower IQ scores Frontal dysexecutive syndrome Apathy Avoidant personality Lack of initiative Social interactions problems Theory of mind deficit Fatigue Sleepiness	50–1000
Late onset/Asymptomatic	>40 years	Mild myotonia Cataracts		50–100
Pre-mutation	N/A	None		38–49

* Note that the described features can be very variable from one individual to another

*N/A, Not applicable*.

Congenital DM1 (CDM) shows a distinct clinical phenotype with distinct clinical features therefore it is not to be considered a severe early form of “classical” DM1. CDM often presents before birth as polyhydramnios and reduced fetal movements. After delivery, the main features are severe generalized weakness, hypotonia, and respiratory compromise. Mortality from respiratory failure is high. Surviving infants experience gradual improvement in motor function, almost all CDM children are able to walk. Cognitive and motor milestones are delayed and all patients with CDM develop learning difficulties and require special schooling needs. A progressive myopathy and the other features seen in the classical form of DM1 can develop although this does not start until early adulthood and usually progresses slowly (Harper, 2001). Clinical myotonia is neither a feature presented in the neonatal period nor can it be disclosed in the electromyogram (EMG). Patients often develop severe problems from cardiorespiratory complications in their third and fourth decades.

The diagnosis of the DM1 childhood onset form is often missed in affected adolescents or children because of uncharacteristic symptoms for a muscular dystrophy and apparently negative family history (Harper, 2001). These patients have cognitive deficits and learning abnormalities (Steyaert et al., 1997) and, as in the congenital cases, degenerative features often develop as these children reach adulthood. There is increasing evidence of early heart conduction abnormalities thus annual electrocardiograms and consideration of electrophysiological studies should be a part of routine management.

The juvenile form with age at onset at 11 years is characterized by school and mating problems and is often under-recognized. These childhood and infantile forms should be considered rather as a CNS disease than a muscular or systemic disease (see Neuropsychiatric Section).

The core features in adult-onset DM1 are distal muscle weakness, leading to difficulty with performing tasks requiring fine dexterity of the hands and foot drop, and facial weakness and wasting, giving rise to ptosis and the typical myopathic or “hatchet” appearance. The neck flexors and finger/wrist flexors are also commonly involved. Grip and percussion myotonia are regular features; however, myotonia affects other muscle including bulbar, tongue, or facial muscles, causing problems with talking, chewing, and swallowing. Elevation of the serum creatine kinase is sometimes present. Cardiac involvement is common in DM1 and includes conduction abnormalities with arrhythmia and conduction blocks contributing significantly to the morbidity and mortality of the disease (Bassez et al., 2004; Chebel et al., 2005; Montella et al., 2005; Russo et al., 2006). In some patients and families, a dilated cardiomyopathy may be observed. Posterior subcapsular cataracts develop in most patients and some patients may develop cataract at an early age without any other symptoms (Garrott et al., 2004). Nocturnal apnoeic episodes and daytime sleepiness are a common manifestations (Laberge et al., 2004). Periodic limb movements in DM1 are also frequently associated (Romigi et al., 2011). Gastrointestinal tract involvement covers irritable bowel syndrome, symptomatic gall stones and gamma-glutamyltransferase elevations. Finally, endocrine abnormalities include testicular atrophy, hypotestosteronism, insulin resistance with usually mild type-2 diabetes. Severe Vit. D deficiency is common in DM1 and it is associated with secondary hyperparathyroidism and primary hyperparathyroidism, though rare may occur. Therefore greater attention should be given to Vit. D status, in order to administer an appropriate replacement theraphy (Passeri et al., 2015).

In late-onset or asymptomatic DM1 patients myotonia, weakness, and excessive daytime sleepiness are rarely present. Before DNA tests became available, there were many examples of incorrect ascertainment, even when using markers such as EMG evidence of myotonia and slit-lamp examination for the characteristic cataracts (Barnes and Hilton-jones, 1994). In late-onset patients, the search for cataracts is helpful for identifying the transmitting person.

**Figure 1:**
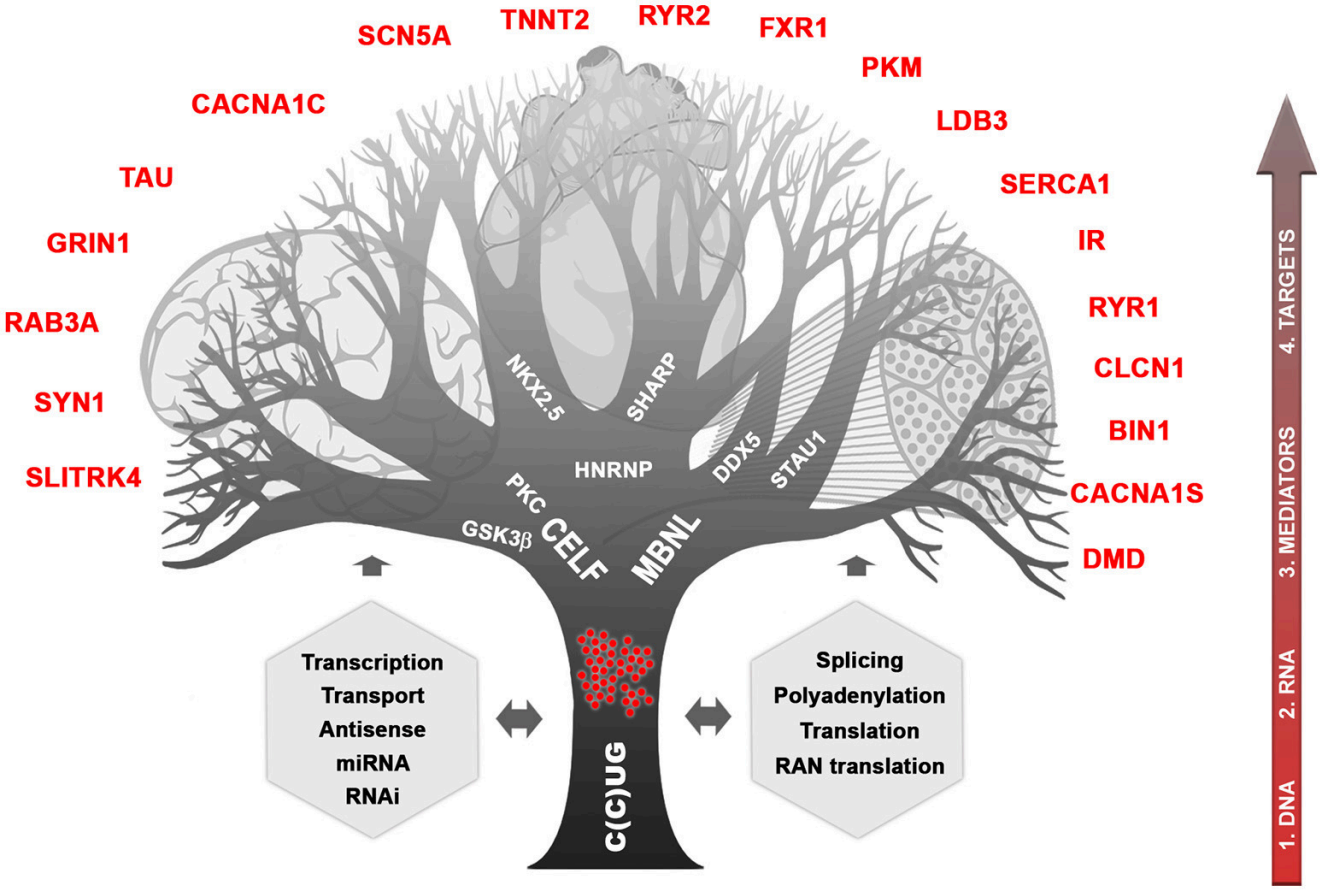
**Complexity of myotonic dystrophies**. The different cellular processes affected by the CTG and CCTG mutations and toxic RNAs are indicated in gray. The possible “mediator” proteins are written in the branches and some of downstream affected genes known so far are named red. The 4 possible entries for therapies are shown on the right side.

About 20 years ago, while studies following the discovery of the DM1 peculiar mutation concentrated mainly on CTG metabolism and direct consequences on neighboring genes, identification of the CCTG expansion in DM2 (in the *CNBP* gene on chromosome 3) helped to provide important milestones to understand how a single mutation can affect so many tissues with such high variability (Liquori et al., 2001). Briefly, the consequences in cascade of CTG and CCTG expansions can be illustrated as a branching tree showing the complex mechanisms involved and the deregulation of many intermediates and final targeted genes (Figure 1). It is clear now that RNAs carrying CUG or CCUG expansions are forming ribonuclear foci easily visible with fluorescent oligonucleotide probes. These foci are trapped into the nucleus of cells expressing the gene involved, during development and in various tissues. They affect a subset of proteins that can be called “mediators” such as MBNL and CELF proteins and others proteins (Pettersson et al., 2015). These mediators are directly affected by RNA foci themselves through direct sequestration (e.g., MBNL) or by triggering stabilization (e.g., CELF stabilized by PKC and/or GSK3ß). In turn, deregulation of the available steady state of mediators disturb various molecular cell processes including transcription, splicing, polyadenylation, mRNA stability, and transport, miRNA and RNAi regulation, conventional and RAN translations (Sicot et al., 2011; Jones et al., 2012; Batra et al., 2014; Kino et al., 2015). In subsequent steps, many other “downstream target genes” will be affected at different molecular levels, which will lead to various cellular processes deregulation in tissues expressing both toxic RNAs and at least part of the mediators. One can predict that severity of mutation toxicity will depend on mutant transcripts levels, on concomitant expression of different mediators through development and in various tissues and finally, on mediators dose-dependency of downstream target RNAs (Jog et al., 2012; Wagner et al., 2016). Regarding CNS, toxic RNA foci are observed in many brain regions, in different cell types and splicing defects have also been reported (Figure 1; Jiang et al., 2004; Caillet-Boudin et al., 2014). This suggests that RNA toxicity and downstream steps involving mediators that can be common with other tissues or specific to brain are also underlying CNS dysfunction in DM patients. Interestingly, MBNL2 appears to be a key actor in DM CNS (Charizanis et al., 2012; Goodwin et al., 2015) but CELF proteins are also very probably involved (Hernández-Hernández et al., 2013; Ladd, 2013; Caillet-Boudin et al., 2014). Nevertheless, little is known about links between the molecular defects and the various symptoms related to CNS. Considered at first sight as muscle and heart disease, growing clinical, neuropsychological, imaging, and histopathological evidence as well as family testimonies demonstrated that DM1 but also DM2, however to a lesser extent, are true brain disorders (Meola, 2010).

## Neuropsychiatric, cognitive, and other neurological symptoms (adults, infants and CDM)

Involvement of the CNS in DM has been first documented in patients with early DM1 onset (congenital or during childhood). CDM (usually >1,000 repeats) is the most severe form of DM1 and may present prenatally with polyhydramnios and decreased fetal movements. At birth, respiratory failure, hypotonia, and feeding difficulties are common. Infants have facial weakness and a characteristic “tented” or “fish-shaped” upper lip (Harper, 2001; Echenne and Bassez, 2013). The mortality rate at birth is high. For the children that pass the critical neonatal period, the developmental profile (especially motor and language skills) improved in early childhood and apparently faster in boys (Prasad et al., 2016). However, moderate to severe intellectual disabilities, reduced IQ-values, speech and language delay, deficit in visuospatial and/or visuo-constructive skills, attention deficit with or without hyperactivity disorder (ADHD), autism spectrum disorder, problems with communication and social anxiety have been reported by several authors in DM1 childhood patients (Angeard et al., 2007, 2011; Meola and Sansone, 2007; Ekström et al., 2008, 2009; Douniol et al., 2009, 2012). Interestingly, a survey distributed to parents of affected child revealed that problems with communication and fatigue appeared to have the greatest impact on childrens' lives (Johnson et al., 2014). It is important to note that in the juvenile form, classical neurological or motor symptoms can be absent and that cognitive (visuo-spatial deficit and executive dysfunction, learning difficulty) or psychiatric symptoms (social mating with peers) can be the only clinical manifestations of the disease. Executive dysfunction reduces initiative, planning abilities and decision making and leads to apathy and inactivity which may have significant influence on quality of life in this cohort of patients.

In adult DM1 patients, cognitive impairment with lower IQ scores is a variable but common feature, even in the least severe form (Jean et al., 2014). Several studies highlight higher–order impairments in executive functions, verbal and non-verbal episodic memory, spatial and visuo-constructive abilities suggestive of a frontal dysexecutive syndrome (Rubinsztein et al., 1998; Modoni et al., 2004; Meola and Sansone, 2007; Sansone et al., 2007). DM1 patients are described as apathetic, with avoidant personality and lack of initiative and they have severe difficulties in daily-living activities including social interactions. Apathy seems to be associated with cognitive status and frontal lobe dysfunction independently of fatigue, sleepiness, age, gender, motor disability, and psychopathological domain (Gallais et al., 2015). This may play role in delay of treatment or diagnosis and studies are ongoing to understand the influencing neuropsychological factors. A large number of DM 1 patients showed reduced ability to recognize facial expressions in recognition tasks and have difficulty in understanding others' mental states from both interaction with others in everyday situations and from their facial expressions. They are impaired in tests assessing the theory of mind (ToM, ability to infer other people's mental states, thoughts and feelings; Takeda et al., 2009; Kobayakawa et al., 2010, 2012; Masaoka et al., 2011; Winblad et al., 2016). In addition, reduced awareness of disease burden is common (Baldanzi et al., 2016a). Beside cognitive and behavioral features, DM1 patients suffer from excessive daytime sleepiness (EDS), sleep apneas, periodic leg movements during sleep. Furthermore, rapid eye movement sleep deregulation and longer habitual nocturnal sleep are reported (Laberge et al., 2009; Heatwole et al., 2012). It has been proposed that EDS is primarily caused by a central dysfunction of sleep regulation while both respiratory muscles (weakness and myotonia) and abnormalities of central control of ventilation contribute to sleep-related disordered breathing (SRDB; Laberge et al., 2013). Fatigue is a major complaint reported by the DM1 patients (Angelini and Tasca, 2012; Heatwole et al., 2012). The origin of this fatigue is complex and can involve both central and peripheral component, fatigue as a sense of tiredness might primarily result from CNS dysfunction (Angelini and Tasca, 2012). These symptoms increase the impact of the disease on social life, and quality of life (Laberge et al., 2009; Heatwole et al., 2012). In a Japanese small cohort of 7 DM1 patients, although DM1 patients had normal olfactory detection (except for 1 out of 7), they exhibited decreased or impaired odor recognition abilities. The loss of odor recognition abilities could be associated with changes in the limbic areas and could represent an interesting biomarker of neurological disease such as DM (Masaoka et al., 2011, 2013). In addition to neuropsychiatric and cognitive symptoms, large epidemiologic studies revealed an increased oncologic risk in DM1 brain, with astrocytoma as the most common subtype (Fernández-Torrón et al., 2016; Gadalla et al., 2016).

Patients with DM2 have also a frontal dysexecutive syndrome with apathy, reduced initiative, and strategic abilities. Visuospatial and executive dysfunctions seemed to be the main cognitive defects, while memory and language impairments appeared in more severe phenotypes. Avoidant personality have been reported but milder in DM2 patients compared to DM1 patients (Meola and Cardani, 2015; Peric et al., 2016). Similar to DM1, fatigue, and impaired sleep or daytime sleepiness are reported among the themes with the highest life impact score (Heatwole et al., 2015). The milder CNS phenotype in DM2 in comparison to DM1 is not well known and may be caused by complex unraveled pathomolecular mechanisms including possible unidentified modifier genes (Meola and Cardani, 2017).

## Imaging features

**Figure 2:**
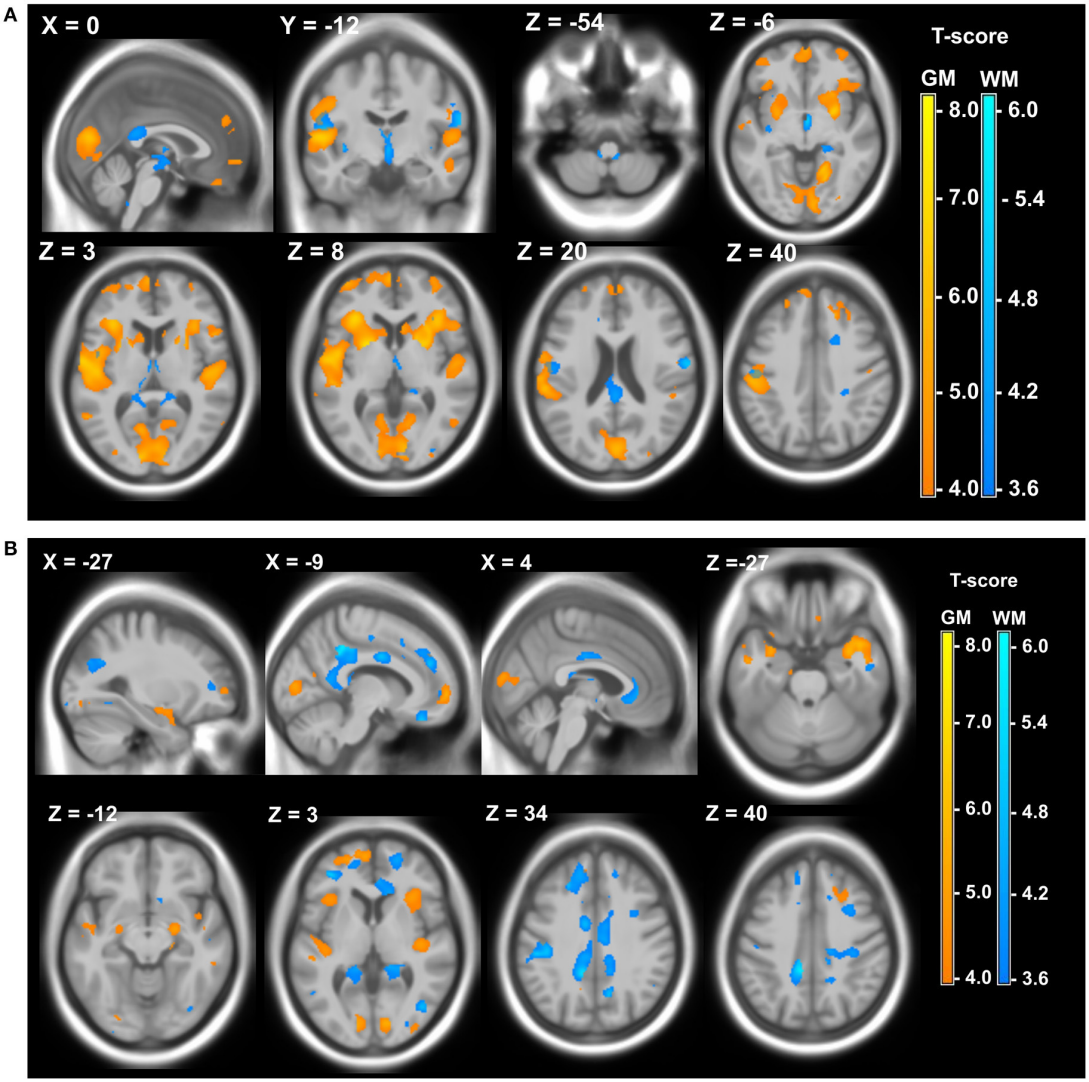
**Local atrophy of gray matter (GM) and white matter (WM) in DM patients. (A)** Local atrophy of GM (yellow) and WM (blue) in 12 DM1 patients compared to 28 healthy controls. **(B)** Local atrophy of GM (yellow) and WM (blue) in 15 DM2 patients compared to 28 healthy controls. Voxelwise multiple regression analysis with group and age as covariates. Age was used as a covariate of no interest. Areas with adjusted *p* at cluster level <0.05 after FWE correction are shown; X, Y, Z: MNI-coordinates: negative X-values reflect left side and positive X-values right sided location. Adapted from Schneider-Gold et al. (2015).

The last 20 years showed a significant increase of different imaging studies both in DM1 and DM2, which confirms the recognition of DM1 as a brain disease. DM1 congenital cases show ventricular enlargement, cortical atrophy and small corpus callosum (Hashimoto et al., 1995; Harper, 2001; Mutchnick et al., 2016). Diffuse T2-hyperintense white matter signals were also reported (Harper, 2001; Bosemani et al., 2014). In young affected children and adolescent, Diffusion Tensor Imaging (DTI) showed significant microstructural white matter abnormalities in frontal, temporal, parietal, and occipital lobar regions. Interestingly, the overall degree of white matter disturbance, was correlated with the working memory deficits (Wozniak et al., 2011, 2014). The observation that in juvenile DM1 patient white matter abnormalities exceeded gray matter strengthen the hypothesis according which developmental changes would be responsible for microstructural white matter alterations in congenital or children cases while gray matter changes would originate from degenerative processes in adult DM1 patients (Caso et al., 2014). In adult DM1 patient, there is also evidence for CNS atrophy significantly correlated with age, possibly indicating progression with time (Baldanzi et al., 2016b). Atrophy appears widely distributed and can be associated with executive dysfunction and with memory and visuo-spatial impairments (Meola et al., 1999; Schneider-Gold et al., 2015; Baldanzi et al., 2016b). Abnormalities in white matter tracts were reported throughout the brain (Harper, 2001; Minnerop et al., 2011; Wozniak et al., 2014; Baldanzi et al., 2016b) as well as dilated Virchow–Robin spaces (DVRS; Renard et al., 2016), reduced cerebral glucose metabolism and cerebral blood flow (Mielke et al., 1993; Annane et al., 1998; Meola et al., 1999, 2003; Romeo et al., 2010) and hypoperfusion (Meola et al., 2003; Romeo et al., 2010). Abnormalities in cerebral metabolites were found in DM1 patients suggesting global neuronal impairment or neuronal loss as well as abnormalities in the glutamatergic system within the frontal lobe of patients with DM1 (Vielhaber et al., 2006; Takado et al., 2015). DM2 patients share some of these features but to a lesser extent (Minnerop et al., 2011; Meola and Cardani, 2015). In addition, structural imaging studies have shown widespread gray matter changes both in DM1 and DM2 patients (Antonini, 2004; Ota et al., 2006; Minnerop et al., 2008; Weber et al., 2010; Schneider-Gold et al., 2015; Serra et al., 2015; Baldanzi et al., 2016b; Zanigni et al., 2016). Gray and white matter local atrophies detected in DM patients using 3T MRI, Voxel-based morphometry (VBM), and Statistical Parametric Mapping (SPM) are illustrated in Figure 2.

## Correlation imaging features/neurological symptoms

Imaging could be a very useful and non-invasive technique for monitoring DM patients during clinical trials. However, strong correlations between observed imaging characteristics and other CNS symptoms must be clearly demonstrated. White matter abnormalities including microstructural damage have been correlated in DM1 patients with cognitive deficits including working memory, processing speed, executive, reasoning, and visuo-spatial impairment, orientation, and attention deficits (Weber et al., 2010; Wozniak et al., 2013, 2014; Caso et al., 2014; Bajrami et al., 2017; Baldanzi et al., 2016b). Sleepiness in DM1 was found inversely associated with white matter fiber integrity (Wozniak et al., 2013). Correlations were found between the sensitivity to facial emotions and frontal, temporal, and insular white matter lesions (Takeda et al., 2009; Kobayakawa et al., 2010). Interestingly, comparison of DM1 and DM2 patients cohorts revealed widespread cortical and subcortical gray matter and white matter atrophy in both types of DM patients especially in the central motor pathways in DM1 and limbic structures in DM2 in correlation with motor dysfunction, cognitive abilities depression, daytime sleepiness, and reduced executive functions (Schneider-Gold et al., 2015; Serra et al., 2015). Furthermore, voxel-based morphometry (VBM) showed bilateral hippocampal decrease in gray matter that correlated to episodic memory deficits in both patient groups (Weber et al., 2010). Transcranial sonography (TCS) studies found pathological brainstem raphe signals associated with excessive daytime sleepiness in DM1 and DM2 patients as well as with fatigue in DM2 patients (Krogias et al., 2014; Peric et al., 2014b; Rakocevic-stojanovic et al., 2016). Functional near-infrared spectroscopy (fNIRS) performed in DM1 patients revealed a correlation between left prefrontal cortex hypometabolism with frontal cognitive performances in DM1 (Caliandro et al., 2013). Changes on brain functional connectivity correlates with atypical personality profiles and Theory of mind (ToM) deficit (Serra et al., 2016a,b). The ToM dysfunctions support the hypothesis that differences in social interactions and personal relationships are consequence of brain connectomics abnormalities and not a reaction symptom. Very interesting is the combining study of cerebral and muscle MRI abnormalities in 10 DM1 (5 congenital and 5 adult) vs. DM2 and controls. A significant difference in the white matter integrity was found in DM compared to controls. In parallel, masticatory muscle volumes were significantly reduced in congenital and adult DM1 compared to controls. All these findings suggest a common mechanism underlying the severity of both muscle and cerebral pathology in DM1 (Franc et al., 2012).

Altogether, the imaging analyses and the attempt to correlate pathological brain changes with cognitive dysfunction or personality profiles suggest that complex neuronal networks involving numerous CNS regions and mechanisms related to plasticity, neurodevelopmental, and neurodegeneration are implicated in myotonic dystrophies.

## Progression of CNS symptoms with age

In order to design future therapeutic trials to target the CNS, it is essential to determine how symptoms change over time. Very recently, two studies have reported for DM1 patients, a cognitive decline on longitudinal studies of 5 or 9 years especially for verbal memory, attention, visuo-spatial construction, and processing speed (Gallais et al., 2017; Winblad et al., 2016). In particular, the Swedish study showed that cognitive decline in adult DM1 correlated with earlier onset and longer duration of the disease suggesting a degenerative process, while the Canadian study supported the hypothesis of a progeroid disease (accelerated and increased aging process). However, these recent studies reinforce the previous observation of Sansone et al who observed a progression of frontal cognitive impairment (attention) both in DM1 and DM2 patients (Sansone et al., 2007). Regarding imaging, recent retrospective MRI evaluation of DM1 patients over 13.4 (± 3.8) years demonstrated that white matter lesions progressed over time although with variable extent depending on the tested patients and families. This suggests a degenerative process of white matter abnormalities in adults DM1 patients (Conforti et al., 2016). Half of the 13 patients of this longitudinal studies showed abnormal ventricular enlargement with time. Unfortunately, brain volumetric techniques were not available at the time of the first MRI observations in this retrospective study. Therefore, more longitudinal studies are needed to accurately determine the level of progression both on white matter and gray matter abnormalities and on more homogeneous cohort of patients (congenital, childhood, juvenile, adult, late-onset). Overall, the interplay between neurological, psychological and social factors can be complex warranting referral to behavioral medicine specialists for neuropsychological testing, evaluation, and treatment. Physicians should remain vigilant of possible substance abuse, child neglect, financial needs, socioeconomic circumstances, personal hygiene, home safety, and unsafe driving. Referral to appropriate social service, local support groups, and organization may be advantageous. There is the current effort to develop care guidelines for patients with myotonic dystrophy that would address the CNS abnormalities as well quality of life.

## Histopathology

A variety of histopathological features have been observed in post mortem DM brain samples. These CNS pathological changes include pre-senile neurofibrillary tangles observed both in DM1 and DM2 brains, Marinesco bodies observed mainly in DM2 and granulovacuolar degeneration (reviewed in Caillet-Boudin et al., 2014). Abnormal tau pathology by immunohistochemistry on brain tissues was found both in DM1 and DM2 (Maurage et al., 2007). Loss of serotonin-containing neurons was observed in relation to hypersomnia (Ono et al., 1998). Heterotopic neurons have been reported in the brain and spinal cord of autopsied DM1 patient with CDM or childhood onset with intellectual deficiency suggesting neurodevelopmental abnormalities (Watanabe et al., 1997). The molecular hallmarks of DM, RNA nuclear foci, are detected in post-mortem DM1 brain tissues as soon as 12–13.5 week (Michel et al., 2015). In DM1 adult post-mortem samples, RNA foci are widely distributed throughout cortical layers, in various brain region, in neurons, astrocytes, oligodendocytes, Purkinje cells as well as in human DM1 iPS or ES-cell-derived neural stem cells (NSCs; Jiang et al., 2004; Denis et al., 2013; Hernández-Hernández et al., 2013). In nuclei, they do not co-localize with PML bodies, nucleolus, perinuclear compartment, or speckles. However, in cortical neurons three components of the proteasome appear recruited by the RNA foci. Moreover, as in muscle cells, RNA foci observed in brain co-localize with MBNL1 and MBNL2 (Jiang et al., 2004).

## DM biomarkers for CNS abnormalities

CNS biomarkers that could be useful for clinical trials were investigated both in blood and cerebrospinal fluid (CSF). Biomarkers of neurodegeneration such as tau and amyloid ß42 protein (Aß42) were investigated in juvenile and adult DM1 patients. A significant decrease of CSF levels of Aß42 was reported as well as increase of the levels of total tau protein (Winblad et al., 2008; Peric et al., 2014a). No correlation between the levels of these CSF biomarkers and neuropsychological impairment were evident. However, they could reflect an alteration of the permeability of the brain blood barrier (BBB) and then represent suitable markers for DM1 patients (Bosco et al., 2015). In addition, increase in CSF IgG, γ-globulin, and possibly in myelin basic protein (MBP) similar to that observed in demyelinating diseases have been reported in DM1 patients (Hirase and Araki, 1984). Interestingly, a decrease of the brain derived-neurotrophic factor (BDNF) was reported recently in the serum of DM1 patients. BDNF is a neurotrophin involved in learning and memory. As it can cross the brain blood barrier, it could be considered as a marker for CNS lesions (Comim et al., 2015).

## Cell and animal models

The development of pathological models is an important step not only to understand mechanisms that link disease mutation and resulting pathophysiology, but also to screen, identify, and test new pharmacological molecules or molecular tools (Plantié et al., 2015). For DM and especially for CNS, a variety of models from cells to mammalian organisms has recently emerged.

Neural stem cells (NSC) derived from Embryonic Stem cells (ES) or from induced Pluripotent Stem Cells (iPSCs) developed in the context of DM1, have shown to recapitulate important features of DM1 (RNA foci, MBNL sequestration, splicing defect; Marteyn et al., 2011; Denis et al., 2013; Xia et al., 2013; Xia and Ashizawa, 2015). DM1 NSC allowed the identification of new cellular pathways and mechanisms affected by the mutation such as decreased expression of members of *SLITRK* family in relation with neuritogenesis and synaptogenesis (Marteyn et al., 2011) and defect in mTOR pathway (Denis et al., 2013). *C. elegans* was one of the first model organisms to be used in laboratory especially to study development, and this is one of the simplest organism with a nervous system. Interestingly, mutation in *C. elegans Mbl1* gene, that shares orthologous sequence with drosophila muscleblind and mammalian muscleblind-like genes induces defect in synapse formation of motor neurons (probably on the presynaptic side) that translates into abnormal locomotion of mutated worms (Spilker et al., 2012). Zebrafish is a simple model organism that has proven to be useful to study neurogenesis and behavior and for large scale forward and reverse genetic screens. Knock-down of *Mbnl2* induced systemic abnormalities including brain malformations (Machuca-Tzili et al., 2011). Expression of (CUG)91 repeat-containing mRNA in embryo resulted in toxicity in the nervous symptoms characterized by delayed development of the head and forebrain structures (Todd et al., 2014). *Drosophila melanogaster* is also a widely used model. CNS development is well-known and multiple useful behavioral tests have been developed. They allow large scale forward and reverse genetic screens (García-Alcover et al., 2014). Different size of CTG repeats have been expressed in flies and CUG repeats over 230 units induce lethality when expressed in the nervous system (Garcia-Lopez et al., 2008; Yu et al., 2011).

Although high throughput screening is not really feasible in mouse models, mice are useful tools to investigate complex cellular and physiological functions in different tissues and during development of a mammalian organism. Disease mouse models are also widely used for preclinical assays, although more than 80% of potential therapeutics tested in mice fail when tested in people (Perrin, 2014). Therefore, animal models for translational research must be characterized in detail and preclinical testing must be proceed with caution. Concerning DM and CNS, few models have been developed so far. They range in two categories that are *Mbnl* knockout mice and transgenic mice expressing CTG repeats in the CNS. *Mbnl* knockout mice have clearly demonstrated the high impact of MBNL proteins (MBNL1 and MBNL2) depletion not only in muscle and heart but also in CNS. Interestingly, comparison of *Mbnl1* knockout with *Mbnl2* knockout and with compound loss of both proteins revealed the major role of MBNL2 in CNS and new developmental functions for these proteins (Kanadia et al., 2003; Charizanis et al., 2012; Batra et al., 2014; Goodwin et al., 2015). *Mbnl2* knockout mice show altered REM (rapid eye movement) sleep regulation, learning and memory deficit associated with decreased synaptic GRIN1 activity and impaired long-term potentiation as well as increase seizure susceptibility (Charizanis et al., 2012). In addition, transcriptomic analyses revealed widespread alternative splicing changes in brain. Despite the preponderant role of MBNL2 in DM CNS symptoms, MBNL1 could also take part in the story. Indeed, *Mbnl1* knockout show mild misplicing events (Suenaga et al., 2012) as well as behavioral changes related to lack of motivation (Matynia et al., 2010), a trait typically observed in DM patients. Furthermore, both MBNL1 and MBNL2 are required for alternative splicing of MAPT exon 2 that is found deregulated in DM patients (Carpentier et al., 2014). The second type of DM mouse model available to study CNS features so far, are transgenic mice such as DMSXL mice (Gomes-Pereira et al., 2007; Hernández-Hernández et al., 2013). These mice express a human *DMPK* transgene carrying >1,000 CTG under the control of its own promoter that allows tissue-specific expression close to that observed in human tissues (Huguet et al., 2012). They display numerous RNA foci in various brain regions associated with brain region-specific mild splicing defects, increase of CELF proteins in frontal cortex and brainstem, increase seizure susceptibility, deficit in short-term synaptic plasticity, anxiety, spatial and working memory impairment, and anhedonia (Charizanis et al., 2012; Hernández-Hernández et al., 2013). In addition, changes in neurochemical levels were observed in frontal cortex and brainstem as well as abnormal expression of synaptic proteins (one of which is also deregulated in *Mbnl* knockout mice) indicative of synaptic dysfunction (Hernández-Hernández et al., 2013). However, better characterization and standardized protocols are needed to determine how these different mouse models can be used for preclinical assays.

## Potential therapeutic strategies targeting CNS

More than 16 years ago, even though the DM1 genetic defect was identified, it remained difficult to propose new therapeutic strategies for DM, as the mechanisms underlying these multisystemic diseases were totally unknown. Since the early 2000s, tremendous progress has been made through identification of the DM2 mutation (Liquori et al., 2001) and development of DM animal models (reviewed in Gomes-Pereira et al., 2011). Since then, new therapeutic tools emerged with aims to target different steps of the DM pathological cascade including (1) the DNA mutation itself, (2) toxic RNAs, (3) mediator proteins, or (4) one of the final targets (Figure 1). Targeting the more upstream as possible should block the beginning of the toxic cascade and correct more defects in tissues. In the context of the multi-systemic DM, the ideal treatment should be systemic and efficient at least in muscle, heart and brain. However, one can imagine also combination of different tissue-specific treatments. The past few years, most efforts have focused on mutant *DMPK* RNA in order to eliminate the toxic RNAs or to release the sequestrated mediators such as MBNL proteins in muscles (reviewed in Klein et al., 2015). Antisense oligonucleotides (ASOs, with various chemistry and/or linked with peptides or nanoparticles) targeting the expanded repeat or specific *DMPK* sequences, small molecules, conjugated cell penetrating peptides (CPPs), miRNA “sponges” and viral-based gene therapeutic approaches have proven to be efficient in cell models and to some extent in animal models especially for muscles (Gao and Cooper, 2013; Klein et al., 2015; Bai et al., 2016; Cerro-Herreros et al., 2016). Twenty-five years after discovery of the DM1 mutation and with these validated proofs of concept, the first phase 1/2a clinical trial was launched in December 2014 using gapmer-ASOs aiming at destroying *DMPK* transcripts in muscles (trial NCT02312011). Beside strategies targeting toxic *DMPK* RNA to release MBNL proteins, CELF proteins are also targets to consider. The inhibition of PKC or GSK3β, proteins known to regulate the phosphorylation of CELF proteins, allowed the reversal of muscle and heart phenotypes in DM1 mouse models (Wang et al., 2009; Jones et al., 2012). CELF proteins (and particularly CELF2) are known to be significant players in the CNS and thus represent important proteins to target, in particular with pharmacological means (Ladd, 2013). A phase 2 trial was recently launched to evaluate the safety and efficacy of tideglusib, a GSK3ß inhibitor (trial NCT02858908). The recent development of genome editing using TALENs or CRISPR-Cas nucleases provides for DM a very appealing new field of investigation (Richard, 2015; Lee et al., 2016; Long et al., 2016). One can dream to be able to remove the deleterious expansion directly on DNA correcting definitively the mutation. Recent studies in yeast, and cell systems have demonstrated the feasibility to remove or reduce CTG repeats with TALEN or CRISPR-Cas nucleases (Richard et al., 2014; Cinesi et al., 2016; van Agtmaal et al., 2017). Furthermore, dual CRISPR-Cas9 cleavages on both side of the CTG expansions in DM1 myoblasts were able to eliminate RNA foci and to restore splicing defects and myogenic capacity (van Agtmaal et al., 2017). However, there is still a long road before to be able to apply nucleases in Human patients and to overcome the difficulties (1) to reach all the tissues affected in DM, (2) to correct DNA in the maximum of cells for restoration of tissue function, and (3) to secure treatments for minimal side-effects.

The current therapeutic developments at RNA or DNA levels should be applicable in principle to the CNS. Therapeutic molecular tools to eliminate toxic RNAs, to release the sequestrated mediators or to removed expanded repeats in the *DMPK* gene should also be efficient in brain cells. This is strengthened by experiments performed in DM1 iPS and DM1 iPS-derived NSC (Xia et al., 2015; Gao et al., 2016). Targeted insertion of polyA signals using TALEN in the *DMPK* gene upstream the CTG repeat expansion eliminated transcripts with expansions, RNA foci and reverse abnormal splicing in the DM1 NSC. The next important challenges for all DM therapeutics approaches will be to face the multi-systemic issue of the disease and to improve efficiency, pharmacokinetics, bio-distribution/delivery/availability, and safety with regards to immune response and gene therapy specificity. Bio-distribution/delivery/availability will be important steps and need to take into account recent finding indicating that cell membranes in DM1 tissues including muscles and possibly heart and brain are not compromised and may keep their barrier capacity (Gonzalez-Barriga et al., 2015). Furthermore, the blood brain barrier (BBB) is an additional barrier to cross before to reach the CNS. Soluble drugs depending on the molecule size and charge might be easier for systemic application and for reaching CNS but they must have high efficacy and low toxicity. Modifications of ASOs are intensively studied especially by pharmaceutical companies to improve cell uptake and bioavailability in tissues, biosystemic distribution, and distribution in the brain via intrathecal injection into the CSF (Geary et al., 2015). For gene therapy, including CRISPR-Cas and TALE nucleases, viral approaches especially adenovirus associated virus (AAV) vectors can be considered although progress has to be made for better targeting and to reduce immune response (Swiech et al., 2015; de Solis et al., 2016; Lee et al., 2016; Ma et al., 2016; Murlidharan et al., 2016). Nanotechnologies as well as cell-penetrated peptides are very promising tools and might help to enable transport across the BBB via intracranial, systemic, or intranasal administration (Krupa et al., 2014; McGowan et al., 2015, 2016; Bai et al., 2016; Kristensen et al., 2016; Sharma et al., 2016; Zhang et al., 2016). Finally, the possibility to modify stem cells open a possible route toward autologous cell transfer (Xia et al., 2015; Gao et al., 2016). Another very important issue will be to target different brain cell types including neurons, astrocytes, and probably others glia cells. More studies are needed to determine the role of each brain cell type in the pathophysiological defects observed in DM CNS. Of course all strategies developed for DM1 will beneficiate to DM2 with transposition to DM2 specificities (CCTG repeat mutation, expression of *CNBP* in which lies the DM2 expansion).

## Conclusion

Although many fundamental aspects of the mechanisms involved in DM remained to be unraveled especially in brain, significant progress has been made in recent years. This covers several aspects that have benefited also from new developments performed in DM muscles and heart. Table 2 summarizes the state of the art for the development of new molecular therapeutic strategies that can be applied to CNS. Starting from the proofs of concept, the development of therapeutic tools to target DNA, RNA, or proteins and high-throughput systems in cells or small organisms to screen for new drugs have already provided relevant candidates (i.e., drugs, ASO, nucleases…). Next barriers to overcome are to improve treatment efficiency, biodistribution, and availability in brain as well as safety. Preclinical mouse models are available and are complementary depending on the therapeutic targets. *Mbnl* Ko mice will be suitable for therapeutic approaches aiming at restoring MBNL functions and transgenic DMSXL can be used for strategies targeting, DNA, RNA, or proteins. Other mouse models may also be helpful, but require further characterization for CNS (this includes conditional mouse models for RNA toxicity, for CELF1 overexpression and *Dmpk* knockin mice, reviewed in Gomes-Pereira et al., 2011). If molecular outputs can be easily defined for both *Mbnl* Ko and DMSXL mice, possible biomarkers traceable in blood or CSF remain to be identified and could give information about possible blood and CSF biomarkers for human DM patients. Behavioral abnormalities have been identified in both models, but practical and easily applicable protocols for preclinical testing should be specified bearing in mind relevance to human DM diseases. For future clinical trials, many recent studies in DM patients have provided finding that will help to determine inclusion criteria, clinical outputs as well as CSF and blood biomarkers. Even though longitudinal studies and attempts to correlate imaging changes with neurological symptoms have emerged significantly, further research on homogeneous cohort of DM1 (congenital, childhood, juvenile, adult, late-onset) and DM2 patients is needed to establish protocols for future trials. All these different issues were discussed during workshops of the international DM-CNS group, reflecting an international effort to combat DM neurological deficits (Axford and Pearson, 2013; Bugiardini et al., 2014; Bosco et al., 2015).

**Table 2:** **State of the art for new DM therapeutic development in CNS**.

**Proofs of concept**	**Drugs**	**ASO**	**Gene therapy**
DNA	**~**	**~**	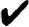
RNA	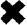	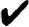	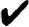
Mediators	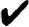	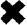	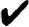
Cells (ES, iPS, NSC)	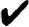	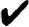	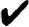
Small organisms	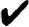	**?**	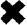
Biodistribution in Mammals CNS	**+/−**	**?**	**?**
**Preclinical feasibility**	***Mbnl* Ko**	**DMSXL**	**Other existing models**
DNA	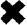	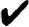	**?**
RNA	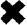	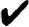	**?**
Mediators	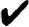	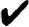	**?**
**Molecular Output**			
RNA foci	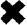	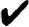	**?**
Splicing defects	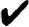	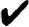 **mild**	**?**
Final target proteins	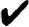	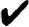	**?**
Histopathology	**?**	**?**	**?**
Tau pathology	**?**	**?**	**?**
**Biomarkers**			
Blood	**?**	**?**	**?**
CSF	**?**	**?**	**?**
**Phenotypes**			
Imaging	**?**	**?**	**?**
Behavior	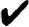	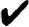	**?**
Sleep	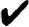	**?**	**?**
Fatigue	**?**	**?**	**?**
**Clinical assay**	**DM1**	**CDM**	**DM2**
**Inclusion Criteria**			
Age	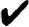	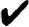	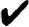
Severity	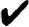	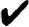	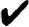
C(C)TG repeat length	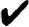	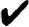	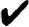
**Clinical Output**			
Imaging	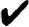	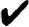	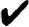
Neuropsy	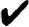	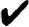	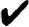
Cognition	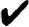	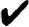	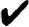
Sleep	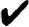	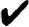	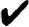
Fatigue	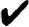	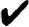	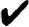
**CSF Biomarkers**			
Ab42	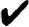	**?**	**?**
Tau	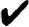	**?**	**?**
other (IgG, MBP…)	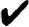	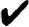	**?**
**Blood Biomarkers**			
BDNF	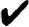	**?**	**?**

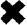
 *Not applicable;* 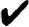 *done or applicable; **?** to be determined;* 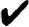 *applicable, must be defined for trials, ~ more or less. Existing symptomatic treatments are not included*.

*ES, Embryonic stem cells; iPS, induced pluripotent cells; NSC, neural stem cells; ASO, antisense oligonucleotides; CSF, cerebrospinal fluid*.

## Author contributions

All authors listed, have made substantial, direct and intellectual contribution to the work, and approved it for publication.

### Conflict of interest statement

The authors declare that the research was conducted in the absence of any commercial or financial relationships that could be construed as a potential conflict of interest.
